# Assessing the health benefits of advice services: using research evidence and logic model methods to explore complex pathways

**DOI:** 10.1111/j.1365-2524.2012.01087.x

**Published:** 2013-01

**Authors:** Peter Allmark, Susan Baxter, Elizabeth Goyder, Louise Guillaume, Gerard Crofton-Martin

**Affiliations:** 1 Health and Social Care Research Centre, Sheffield Hallam UniversitySheffield, UK; 2 Section of Public Health, School of Health and Related Research, University of SheffieldSheffield, UK; 3 Citizens AdviceLondon, UK

**Keywords:** health inequalities, logic model, poverty, primary care, social determinants of health, welfare benefits

## Abstract

Poverty is positively associated with poor health; thus, some healthcare commissioners in the UK have pioneered the introduction of advice services in health service locations. Previous systematic reviews have found little direct evidence for a causal relationship between the provision of advice and physical health and limited evidence for mental health improvement. This paper reports a study using a broader range of types of research evidence to construct a conceptual (logic) model of the wider evidence underpinning potential (rather than only proven) causal pathways between the provision of advice services and improvements in health. Data and discussion from 87 documents were used to construct a model describing interventions, primary outcomes, secondary and tertiary outcomes following advice interventions. The model portrays complex causal pathways between the intervention and various health outcomes; it also indicates the level of evidence for each pathway. It can be used to inform the development of research designed to evaluate the pathways between interventions and health outcomes, which will determine the impact on health outcomes and may explain inconsistencies in previous research findings. It may also be useful to commissioners and practitioners in making decisions regarding development and commissioning of advice services.

## What is known about this topic

- Poverty is positively associated with poor health.
- Research to date has reported that advice services lead to financial gain, but no direct evidence of physical health improvements and limited evidence of mental health improvements.

## What this paper adds

- A causal pathway between welfare interventions and health and well-being improvements can be constructed from the available evidence. By identifying key elements in a causal pathway via a logic model, research can be used to identify plausible ways in which an intervention improves health and also where the gaps in evidence lie.
- Logic models can be used to help illuminate complex pathways from interventions to outcomes, which may be of benefit to both service planners and researchers.

## Introduction

In the UK, some commissioners of local health services have pioneered the provision of advice services as part of community and primary care. These advice initiatives have been funded in the expectation that such social interventions might be expected to improve recipients’ health. The literature supports this reasoning, research clearly indicating that poverty is associated with ill-health. A recent report commissioned by the UK Government amassed evidence of a social gradient in health, with those with the lowest socioeconomic status having poorest health and a gradient to those at the highest socioeconomic status having the best health (Marmot 2010). It might be thought therefore that improvements in individual income would be associated with improved health. However, reviews of evidence (e.g. Adams *et al.* 2006) found no measured effect on physical health from improved welfare benefit income (which is almost always delivered to those with lower socioeconomic status). Authors of these reviews emphasise that this is an absence of evidence for the effect, rather than evidence for absence of effect. This lack of evidence may be the result of complexity, with significant challenges in establishing a clear causal pathway between intervention and health outcome.

Logic models (also known as impact or conceptual models) originate from the field of programme evaluation, and are typically diagrams or flow charts that convey relationships between contextual factors, inputs, processes and outcomes (Joly *et al.* 2007, Anderson *et al.* 2011). They are designed to read from left to right illustrating pathways between inputs, strategies, outputs, and short-term, intermediate and longer-term outcomes. Logic models can provide a visual means of examining complex chains of reasoning and can be valuable in providing a ‘roadmap’ to illustrate influential relationships and components between inputs and outcomes (Schmitz 1999, Kellog Foundation 2004). Most models described in the literature are developed via consultation exercises with experts in the field and are thus open to criticisms of being unsystematic and biased. Recent work by the team has, however, demonstrated the possibility for models to be constructed drawing on systematic review techniques (Baxter *et al.* 2010). The study reported here aimed to use these methods to explore available evidence regarding the potential impact of advice interventions on health outcomes.

## Methods

This work used an innovative combination of logic model methods synthesising data collected using the underlying principles of the systematic review process. The research question for the review was: what are the elements in a causal pathway between advice interventions and health outcomes?

### Inclusion criteria

The review searched for published peer-reviewed international papers and grey literature from the UK, with broad study design inclusion criteria to maximise the range of work encompassed in the synthesis and to underpin development of the logic model. Research published in English up to February 2010 was eligible for inclusion; the start date was set *de facto* by the time span of the databases searched. Designs included were intervention studies, quantitative work reporting associations, qualitative studies, systematic reviews, literature reviews and discussion papers. Papers describing links between any type of advice intervention delivered in any setting, to any population, and including all forms of outcome measures and evaluation were considered. In addition to the peer-reviewed and grey literature, we sourced local and Welsh and English data from UK Citizens Advice; no Scottish data were available.

### Search strategy

For the first wave of the process, relevant published literature was identified via searching of the Medline, Embase, HMIC PscyINFO, SCI and SSCI, CINAHL, ASSIA, LISA, Sociological Abstracts, Cochrane Library, EPPI Centre and Google Scholar electronic databases. Search terms used were variants of citizens advice, advice and citizens advice bureau (the CAB does not use an apostrophe for the term ‘Citizens’).

### Selection of studies for review

**Figure 1:**
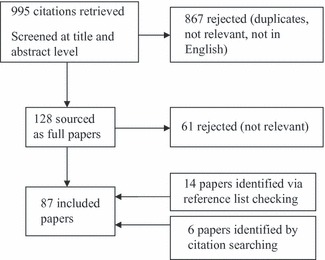
Flow chart illustrating the process of inclusion and exclusion.

The initial searches retrieved 995 citations, which were screened at title and abstract level. One hundred and twenty-eight citations appeared relevant and were retrieved as full papers. The sifting and selection of papers for inclusion were carried out by two members of the research team. In addition to database searching, the reference lists of included papers were examined; there was citation searching of key papers, and experts in the field were contacted to suggest any further references. Figure 1 provides an illustration of the identification process.

### Data extraction and synthesis

**Figure 2:**
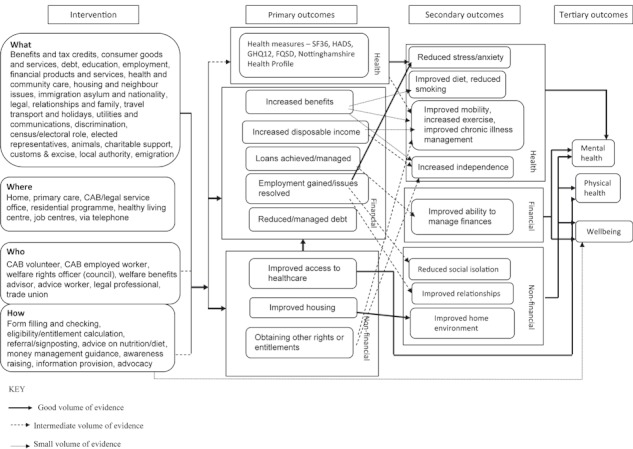
Potential links between advice interventions and health outcomes.

Papers meeting the inclusion criteria were read and data extracted using a standardised extraction form encompassing author/date, details of any intervention, measures used, reported outcomes linked to health and/or well-being, and links to other non-health impacts. See online Appendix S1 for a summary of individual studies. Following extraction, data from each column were examined by the research team and the logic model was built column by column underpinned by the evidence (Figure 2). So, for example, the first column (the intervention) was expanded by synthesising elements of interventions described across the set of papers. The second column (primary outcomes) was developed by synthesising reported study measures and outcomes, and so on. The primary outcomes were the direct aims of the intervention, such as obtaining unclaimed benefits. We defined secondary outcomes as those (i) with evidence of causal links to the intervention via the primary outcomes reported in the literature and (ii) with already established links to health or well-being.

### Quality appraisal

In contrast to standard systematic review methods, we set no quality standards on the papers reviewed other than their publication in peer-reviewed journals. We did, however, account for volume of evidence in our logic model. This has three standards, shown by differences in line thickness. The thick line indicates the greatest volume of evidence, for example, quantitative evidence from large-scale epidemiological studies. The thicker dotted line indicates less volume of evidence, for example, from before-and-after data. The thinnest dotted line indicates a low level of evidence, for example, from one or two small-scale qualitative studies.

## Results

Our searches identified 87 documents that met the criteria for inclusion. Table 1 categorises the documents reviewed by study design. Data from the included documents were used to construct the logic model, describing components of an advice intervention, the short-term or primary directly measured outcomes, the secondary or more indirect benefits following the intervention, and finally potential links between these outcomes and long-term improvement in health and well-being (see Figure 2). Some examples of the evidence underpinning each component of the logic model are outlined below; detailed information is in the online Appendix S1 (Summary Table).

**Table 1:** Summary of the included literature by study design

Design	No.	Papers
Mixed method	17	Abbott and Abbott (2007), Caiels and Thurston (2005), Clarke *et al.* (2001), Coppel *et al.* (1999), Doncaster (2008), Finch *et al.* (1993), Galvin and Sharples (2000), Gillespie *et al.* (2007), Lishman-Peat (2002), Marcella and Baxter (2000), Memel and Gubbay (1999), Middleton *et al.* (1993), Nosowska (2004), Reading *et al.* (2002), Sanderson and Mahon (2003), Sherr *et al.* (2002), Sherratt *et al.* (2000)
Cross-sectional	26	Abbott and Hobby (2000, 2003), Ambrose and Stone (2003), Atkinson *et al.* (2006), Beer *et al.* (1998), Borland and Owens (2004), Buck *et al.* (2008), Fruin and Pitt (2008), Greasley (2003), Greasley and Small (2005a), Harding *et al.* (2002, 2003), Hobby and Emanuel (1998), Hoskins and Smith (2002), Hoskins *et al.* (2005), Jenkins *et al.* (2008), Langley *et al.* (2004), Levy and Payne (2006), Paris and Player (1993), Pleasence and Balmer (2009), Pleasence *et al.* (2007), Powell *et al.* (2004), Smith and Patel (2008), Taylor *et al.* (2009), Toeg (2003), Veitch and Terry (1993)
Discussion papers	11	Abbott (2002), Andersen (2009), Anyadike-Danes (2010), Anyadike-Danes and McVicar (2008), Citizens Advice Bureau (2010), Cullen (2004), DeSouza *et al.* (2007), Ennals (1993), Jacoby (2002), Moffatt and Mackintosh (2009), Porter (1998)
Longitudinal	7	Hobby (1999), Abbott and Davidson (2000), Abbott and Hobby (2002, 2003), Abbott *et al.* (2005), Campbell *et al.* (2007), Jones (2009)
Qualitative	12	Craig *et al.* (2003), Day *et al.* (2008), Greasley and Small (2005b), Moffatt *et al.* (2004, 2010), Moffatt and Scambler (2008), Moffatt and Higgs (2007), Moffatt and Mackintosh (2009), Moffatt, Mackintosh (2006), Popay *et al.* (2007), Turley and White (2007), Winder *et al.* (2008)
RCT	3	Burns *et al.* (2007), Mackintosh *et al.* (2006), Pleasence and Balmer (2007)
Systematic review	11	Adams *et al.* (2006), Bambra *et al.* (2010), Connor *et al.* (1999), Dobbie and Gillespie (2010), Dowling *et al.* (2003), Greasley and Small (2002), Hanratty *et al.* (2008), Hoskins and Cater (2000), Lucas *et al.* (2008), Wiggan and Talbot (2006), Williams (2004)
Total	87	

### Components of an advice intervention

In the model, we have divided the intervention into four components: who delivers it, where it is delivered, what is delivered (the content of the intervention) and how (methods/format of delivery).

### Primary outcomes

Short-term (primary) outcomes described in the included papers related to (i) health measures, (ii) financial gain and (iii) non-financial benefits.

#### Health

Fifteen papers used validated tools to assess whether there was a measurable impact of advice services on health (Memel & Gubbay 1999, Abbott & Hobby 2000, 2002, 2003, Greasley 2003, Langley *et al.* 2004, Powell *et al.* 2004, Abbott *et al.* 2005, Caiels & Thurston 2005, Mackintosh *et al.* 2006, Campbell *et al.* 2007, Pleasence & Balmer 2007, 2009, Jones 2009, Taylor *et al.* 2009). Only one paper (a review of evaluations of small UK-based debt advice initiatives) reported evidence of physical health gains; this related to the avoidance of stress-related problems (Williams 2004). No difference was found in the use of NHS services following the intervention (Abbott & Davidson 2000, Abbott & Hobby 2002). In contrast to the lack of quantitative evidence regarding direct physical health benefits, some qualitative data showed that recipients perceived that their physical health improved as a result of receiving additional income (for example, Clarke *et al.* 2001, Greasley 2003, Borland & Owens 2004, Greasley & Small 2005a,b, Moffatt *et al.* 2006a, 2010, Doncaster 2008, Moffatt & Scambler 2008).

In relation to mental health and emotional well-being, the most recent review (Adams *et al.* 2006) reported a statistically significant improvement in mental health measures, particularly depression, although one controlled trial did not find a positive mental health effect from debt advice (Pleasence & Balmer 2007).

#### Financial

The most commonly reported financial benefits arose from unclaimed benefit income and from help with managing debt. A 2006 systematic review found that the usual financial outcome was a lump sum followed by an increase in recurring benefits (Adams *et al.* 2006).

#### Non-financial

Five papers reported non-financial effects following an advice intervention (Reading *et al.* 2002, Ambrose & Stone 2003, Moffatt *et al.* 2004, Mackintosh *et al.* 2006, Doncaster 2008). Material benefits included free prescriptions and dental treatment, council tax exemption, respite care, meals-on-wheels, disabled parking permits, aids and adaptations around the home, help with energy use and a Community Care Alarm scheme. Wider problems addressed fell into categories of housing, employment and relationships.

### Secondary/indirect outcomes

We defined secondary outcomes as those (i) with evidence of causal links to the intervention via the primary outcomes reported in the literature and (ii) with already established links to health or well-being. For example, if data show that provision of a parking permit results in increased mobility for recipients, we recorded this as a secondary outcome. The secondary outcomes of interest in our model were those that either constituted or plausibly contributed to health and well-being. As with primary outcomes, we have used the categories: health, financial and non-financial.

#### Health

The literature widely discusses links between financial difficulties, stress and illness (e.g. Jacoby 2002) and hence interventions to tackle these problems might be expected to reduce financial stress and improve health. An additional indirect impact may be due to a counselling effect, that people felt their health improved as a result of being listened to (Veitch & Terry 1993, Lishman-Peat 2002, Moffatt & Mackintosh 2009).

A number of papers provided data reporting what recipients said they did with increased income and some of these changes in spending may be linked to health benefits (Paris & Player 1993, Moffatt *et al.* 2004, Nosowska 2004, Adams *et al.* 2006, Levy & Payne 2006, Moffatt & Higgs 2007, Turley & White 2007, Doncaster 2008, Andersen 2009, Moffatt & Mackintosh 2009). Craig *et al*. (2003) categorised these uses as increased spending on essentials such as food; spending to increase mobility, such as taxis; the provision of additional goods and services such as gardeners; spending on large household items such as fridges; and spending on personal items such as presents for grandchildren. Improved mobility was also reported as an outcome of some of the other non-financial benefits such as disabled parking permits and adaptations to the house (Paris & Player 1993, Abbott & Hobby 2003, Greasley 2003, Toeg 2003, Caiels & Thurston 2005, Moffatt & Scambler 2008, Moffatt & Mackintosh 2009, Moffatt *et al.* 2010). Provision of meals-on-wheels services, for example, may have potential to improve diet (Craig *et al.* 2003).

#### Financial outcomes

Taylor *et al.* (2009) showed a strong association between what they term ‘financial capability’ (the ability of an individual to manage his/her money) and psychological well-being, such that changes in the former directly correlate with changes in the latter. Greater incapability is associated with stress and increased reporting of mental health problems, particularly depression. If an intervention can improve individuals’ financial capability, it is likely that mental health benefits will follow.

#### Non-financial outcomes

Other outcomes secondary to the direct impacts of advice services include reduction in social isolation (Moffatt *et al.* 2004), improvement in family and other relationships (Dobbie & Gillespie 2010) and improved home environment (Connor *et al.* 1999, Abbott 2002).

### Health and well-being

Following construction of the first three columns of the logic model from the included data, we further examined the papers for evidence of links from reported outcomes to long-term health and well-being benefits. We were unable to find evidence underpinning these plausible chains of reasoning in these papers and therefore conducted further searching across the wider literature, using search terms relating to debt, employment, disposable income, housing, health-care, mental health, physical health and well-being to identify further evidence for this final step in the causal pathway between intervention and health outcomes.

This additional searching was able to locate papers that supported associations between many of the intermediate outcomes identified in the advice literature and longer term impacts on health and well-being. Studies linked improved housing and mental health benefits (Rymill & Hart 1992, Hopton & Hunt 1996, Glover 1999, Blackman & Harvey 2001, Peace & Kell 2001, Migita *et al.* 2005, Ho *et al.* 2007, Egan *et al.* 2010, Liddel & Morris 2009). Also, evidence was found supporting a relationship between improved housing and physical health gains (Willis 2007, Roman *et al.* 2009, Bambra 2010).

## Discussion

The logic model synthesises evidence of plausible routes to link welfare interventions to health benefits. Previous systematic reviews have been unable to demonstrate evidence of clear health gain. One explanation may be that the research thus far has been of limited quality. Our search of the literature confirms that there has been little empirical work that is controlled or longitudinal. The lack of studies with long-term follow-up is important as physical health benefits might take time to emerge following an intervention and thus be unreported in available work. Another potential explanation for lack of evidence of effect may be that the tools used have not been sufficiently sensitive to detect change or may not be measuring outcomes of importance (Moffatt *et al.* 2006b, Moffatt & Mackintosh 2009). In the papers included in this review, the quantitative measures used were largely unable to detect any change in health status. Many of the qualitative studies, however, suggested that people believed that their health had improved, which may significantly impact an individual’s well-being.

A further obstacle to demonstrating a positive impact on health relates to countervailing forces in the population, such as a steeper than average trajectory of decline for the group entitled to advice (Abbott & Hobby 2000, 2002, 2003, Turley & White 2007). Therefore, the demonstration of significant positive effects using standard baseline and outcome measures presents considerable challenges.

A further possible reason for the failure to find an effect is conceptual. RCTs and other trial designs focus on input and output. This has been termed a ‘black box’ view of mechanisms (Williams 2003, Connelly *et al.* 2007). This often works well with closed systems, such as human bodies and drugs; however, it is problematic with open systems, such as societies. Logic models, in contrast, take a systems approach and are able to portray elements and relationships within a system (Andersen 2009). The model developed here identifies how the intermediate outcomes set in train by advice services can lead towards improved health for its recipients. For example, there is evidence that financial benefits, such as disability allowance, added to non-financial benefits, such as disabled parking permits, improve people’s mobility. From other sources, we know that improved mobility improves physical and mental health. Linking these factors in a model conceptually, we can describe the pathway from financial benefits to improved mobility and to a positive effect on well-being.

The largest proportion of the evidence we identified related to positive financial outcomes following advice interventions. This primary outcome was then most commonly linked to the secondary outcome of an improvement in mental health. In particular, the literature reported a strong link between a reduction in debt and reduced stress or anxiety. We explored the potential to differentiate strength of evidence by using different thickness (or weighting) of the connecting arrows, using study design type or quantity of evidence as indicators of strength. The model we have developed includes these arrows; however, we have concerns that this may indicate only where links may be more feasible to demonstrate in empirical work, rather than representing true strength of relationships. The further development of methods for differentiating evidence in logic models is an area worth exploring in future studies.

This work may be criticised for departing from standard systematic review methods in a number of ways relating to quality appraisal by including diverse sources of evidence, treating study designs as equal and not carrying out a critical evaluation of included papers. A standard review would have excluded much of the literature sourced here. We would argue that while quality criteria and likelihood of bias in study design are key aspects to consider, the building of the logic model was strengthened by drawing on all available literature (cf. Dorwick *et al.* 2009). As described above, questions of quality were considered in development of the model linkages. The type of included evidence must be considered, however, in drawing conclusions from this review. In terms of the review itself, our use of terms related to ‘advice’ might have resulted in the exclusion of relevant material from countries in which this term does not have the welfare implications it evidently has in Anglo-American contexts.

This evidence-based logic model provides a framework to inform both researchers and practitioners. The model illuminates the complexity of elements at all phases of a causal pathway from intervention to long-term impacts on health and well-being.

For practitioners, the model has at least three uses. First, it provides a graphic representation of where evidence has been reported for associations between elements. This is useful to support decisions regarding service provision. Second, it may be used to inform decisions regarding the type of provision to fund. Third, it may help practitioners to identify and develop linkages within existing services. The finding of some indications that those with fewer financial concerns might smoke less, for example, might encourage practitioners to combine the offer of a welfare benefits check-up with a stop-smoking service.

For researchers, the model indicates where research might be directed to test the causal chain. Currently, the link between advice and financial outcomes is well demonstrated, with further work required to investigate other relationships in the proposed model. An economic modelling approach to examine financial outcomes in relation to intervention costs would, however, be helpful. We believe that the model identifies the range of variables and potential outcomes that should be considered in any studies, and provides a framework for future research.

## Notes

**Appendix S1** Summary table [for publication on web].
