# Supplementary material for: Assessing the health benefits of advice services: using research evidence and logic model methods to explore complex pathways
Source: Health Soc Care Community. 2013 Jan;21(1):59–68. doi: 10.1111/j.1365-2524.2012.01087.x (PMC3557712; doi:10.1111/j.1365-2524.2012.01087.x)
Supplement: Supplementary file 1 [file hsc0021-0059-sd1.doc]

**ON-LINE APPENDIX: Summary table [For publication on Web]**

| **KEY** |  |
| --- | --- |
| AA | Attendance Allowance; a welfare benefit related to disability |
| BHPS | British Household Panel Survey - a longitudinal survey of UK households looking at a wide range of indicators, including health |
| BMI | Body Mass Index - used as an indicator of healthy (or unhealthy) weight |
| DLA | Disability Living Allowance; a welfare benefit related to disability |
| EQ / EQ5D | Standardised measure of health status developed by a European (E) Quality of Life (Q) group based on 5 dimensions [mobility, self-care, usual activities, pain or discomfort, and anxiety or depression] |
| GHQ | General Health Questionnaire |
| GP | General Practitioner |
| HAD/ HADS | Hospital Anxiety and Depression Score |
| HAQ | Health Assessment Questionnaire |
| IB | Incapacity Benefit; a welfare benefit related to disability |
| MYMOP | Measure Yourself Medical Outcome Profile; a health survey tool |
| NHP | Nottingham Health Profile: a health survey tool |
| NHS | National Health Service |
| PCT | Primary Care Trust; an organisational unit within the NHS |
| RCT | Randomised Controlled Trial |
| SF36 | Short Form 36; a health survey tool |

| Author/date  Type of document | 1) The intervention | 2) Short term outcomes/measures | 3) Link to health/wellbeing | 4) Link to other impact |
| --- | --- | --- | --- | --- |
| Abbott & Hobby 2000a  Mixed method evaluation Follow Up 6 months and 12 months | Income maximisation advice, Health and Advice Project, Citizens Advice Bureau managed scheme delivered in health centres  Intervention delivered by paid workers | Participant perceptions  SF36 | Of the 48 who reported increased income as a result of the service, 79% said that a long term illness had become worse at 6 months Follow Up and 44% had developed a new illness, 31% reported increased stress.  Users had poor health, typically chronic and often deteriorating therefore improvements in health would not be expected.  Of the 48 who reported increased income 60% felt that the improvement in income had affected how they felt about life and/or that their health had improved.  For those with increased income - On SF36 sig increase in vitality, role functioning –emotional and mental health. No change at 12 months. No significant change in physical health. |  |
| Abbott 2000b  Mixed method | Liverpool - Primary Care Deprivation Initiative (PCDI)  Primary health care teams refer patients to non-professional PCDI support workers who offer support, information and advocacy. |  | No statistically significant change in NHS usage -  Qualitative data showed satisfaction with service |  |
| Abbott & Hobby 2002  Mixed method evaluation of service users receiving benefits advice from seven research sites across England (Jan 2000 to March 2001) |  | SF 36  Review of health records on health service usage before and after | SF36 vitality and Mental Health scores increased for those whose income increased (stat significant) - 6 months  Improvements were sustained but reduced at 12 months  Those who had to wait for benefits had disadvantage to health  Comparison with control group impossible as many GPs did not grant access to the data: however, no decrease in activity noted for the intervention group so no indication of improved health here | 178 received increased income  NB Beware distortion - those refused benefits might be more healthy in general than those who are granted them - making comparison difficult. |
| Abbot 2002  Discussion | Welfare benefits advice |  | Elements in pathways from poverty to ill-health are proposed – impact of material factors (either individual or environmental) on health and/or impact of psychological factors (either individual or environmental) on health. Individual material factors may be lack of basic needs or health-damaging behaviours. Environmental factors may be features such as pollution/danger or absence or inadequacy of services.  References for impact of inadequate housing on health.  Reference for smoking reduction following welfare payments.  Benefits may enable access to services (references)  Effect on health would be more powerful in younger clients | Limitations regarding how income increases can impact on physical health, welfare benefits advice may therefore impact more on psychological rather than physical health.  Unemployment and work insecurity linked to poorer health.  Financial strain linked to onset and maintenance of mental illness.  Whitehall study – stress and physiological illness  Health damaging behaviours due to increased stress e.g. smoking  Social inequalities rather than social conditions cause ill health (Wilkinson) |
| Abbott 2007  Cross-sectional characteristics of service users | Citizens Advice Bureau users seeking employment advice |  |  | Workplaces users originated from were predominantly non-union, mix of small and large firms. Heterogeneity in terms of age, salary and qualifications. |
| Abbott & Hobby 2003  Longitudinal study | Advice workers in Citizens Advice Bureau services within GP practices. 90% reported that they went seeking welfare benefits advice. 85% received advice about income, 71% advice regarding a new benefit, 53% related to DLA. Table indicating type of advice received. | Participant perceptions  SF36 | Three quarters reported feeling less anxious and/or worried after the consultation.  Those seeking the service were substantially less well than the population as a whole (SF36 scores + self report) |  |
| Abbot et al. 2005  Longitudinal study Follow Up 6m and 12m | Advice workers in GP surgeries providing welfare benefits advice only, clients referred elsewhere for help with other needs more | 1.SF36 health profile instrument; 2. Self-report of any claims/appeals made and any income increase; 3. Self-report regarding how any income increase had changed their daily living; | Increased income sig associated with reduction in bodily pain – other measures no difference.  At 12 months small improvement in mental health and emotional role  Over a third could now eat more and/or better food  SF36 improved for both groups at 6 months. | Only a third reported spending increased money on food or fuel; Over half better able to pay bills; Nearly half used transport |
| Adams et al 2006  Systematic review | Welfare rights advice delivered in healthcare settings  30 (55%) of advice was delivered by employees or volunteers of Citizens Advice Bureau; 22 (40%) advice mainly delivered by welfare rights officers, usually identified as employees of local government. Referral was mainly from member of primary care team, other relevant agency, self-referral or a combination (32 - 73%). Eligibility to received advice varied from all, to selected sub-groups based, for example, on age. | Health outcomes were measured using a variety of measures, primarily SF36, HADS, MYMOP, and NHP. There was also qualitative assessment. | Sample sizes were small and time period short so evidence from quantitative data on physical health was absent; there was some evidence of improved mental health.  In the quantitative results, occasional significant correlations were found but the main impression and overall result is of no impact. The qualitative results were more positive. | There is strong evidence of improved financial outcomes usually in the form of a lump sum followed by an increase in recurring benefits; the total in the first year averaged £1026. In many studies, cases were pending. |
| Ambrose & Stone 2003  Mixed method evaluation | Citizens Advice Bureau service  10% of clients are seen by specialist services (benefits, consumer, employment, other) | Financial outcomes, user perceptions of gain | 4000 of 15000 enquiries related to benefits, 3000 to consumer debt, 1900 housing. Additional income in regard to benefits £435,000, employment 125,000, consumer including debt 50,000.  Outcomes described – extra recreational visits, buying extra non-prescribed medication, extra and better food, private physiotherapy session, greater peace of mind, fund alternative therapies | Outcomes described – money to travel (places of interest, visit family), increased confidence, better family relationships, able to budget, independence and reduced feeling of being a burden, can pay others to do jobs, money to pursue social, creative and therapeutic activities |
| Anderson 2009  Cross sectional analysis - 14 waves of the British Household survey | No intervention detailed | GHQ12 | Those in the middle group experience the biggest negative change in subjective wellbeing as against the high or low social classes.  Focus is on the effect by class on health and wellbeing of being made unemployed. | Argues for targeting help for the newly unemployed on the poorest. |
| Anyadike Danes 2010  Discussion |  | Incapacity benefit claims |  | IB is claimed by far greater proportions in some areas, such as Wales and NE England, than others, such as SE England. Further, there are differences in the reason for claims; physical problems being predominant in the high claimant areas; these predominate also in SE but mental health issues are more common than in high claim areas. |
| Anyadike Danes 2008  Discussion | No intervention detailed | Incapacity benefit claims | Why have IB claims risen whilst health of population improves? Paper only partially answers this; but the early 90s saw influx onto IB; since then this has reduced but the length of stay has increased. | Main conclusion is that neither country respects doulia rights: i.e. the unpaid work of carers is reciprocated by arrangements of support such as flexible working rights and by carer benefits; however there are important differences e.g. Netherlands is more generous in payments. |
| Atkinson 2006  Cross-sectional study |  | Questionnaire identifying people's relative strengths and weaknesses in four financial capability domains: managing money; planning ahead; choosing products; and staying informed. The questionnaire also had a money quiz. |  | Model of financial capability  Five groups of respondents were identified, from "No weak areas" to "Five weak areas". Analysis of each group showed that, for example, the "Five weak area" group included those on low incomes and young. This group analysis could be useful in other work analysing Citizens Advice Bureau customers. |
| Bambra 2010a  Review of reviews of interventions based on social determinants of health | Interventions categorised by: water and sanitation; agriculture and food; access to health and social care services; unemployment and welfare; working conditions; housing and living environment; education; and transport. |  | Best evidence for reduction of health inequality is in the area of housing and the work environment. Two of the nine reviews in housing concerned the effect of rental assistance programmes (Acevedo 2004, Anderson 2003). The evidence on unemployment and welfare includes the Adams review and nothing else of relevance; nothing relevant in the category of health and social care either - the authors note that no reviews of economic access (affordability) to health care were identified. |  |
| Beer et al. 1998  Survey evaluation of Citizens Advice Bureau service Shetland Islands | Citizens Advice Bureau service  52 (of 146) initial contacts made by phone. 85% of clients female. Categories of contact – benefits, consumer, employment, housing, legal, relationship, tax, utilities, community care, education, health, immigration, nationality, misc, signposting. Signposting most frequent followed by benefits, then housing, legal problems, relationship, community care |  |  |  |
| Borland & Owens 2004  Survey evaluation of service via users and GPs | Citizens Advice Bureau services in GP surgeries, community hospitals, in people’s own homes, a few Citizens Advice Bureau premises, delivered by paid specialist and volunteer generalists | GP and user views | 62% of GPs agreed or strongly agreed that the service improved general health, 61% that it helps patients deal with chronic illness | 88% of users reported that they felt better after seeing the advice worker  84% of GPs agreed or strongly agreed that the service gave users a feeling that someone cares, 84% that it reduces feeling of hopelessness, 68% that it gives people a lift, 63% that it increases self esteem, 77% that it increases quality of life |
| Buck et al. 2008  Cross sectional analysis of Social Justice Survey regarding how legal issues are dealt with | Table showing percentage of successful advice received by problem type and advisor type including CAB, solicitor, trade union, local council, police, insurance company, doctor/health care worker |  |  | Some social and demographic differences in tendency to do nothing rather than obtain advice eg those with motorised transport less likely to do nothing. Those in receipt of benefit had low rate of inaction and high percentage seeking advice. Highest level of inaction among those without any qualifications. Reasons for inaction varied significantly by problem type. |
| Burns 2007  RCT – mental health patients in six European countries | Individual placement and support (IPS) (aka place and train) or vocational services (aka train and place). | Entering competitive employment. |  | IPS group did better in regard to employment |
| Caiels & Thurston, 2005  Mixed method evaluation of Warrington Citizens Advice Bureau project | Citizens Advice Bureau GP outreach service | Pre and post service use questionnaire, SF12, views via interviews | 78% reported feeling less anxious after seeing the Citizens Advice Bureau adviser, 2% said they felt more anxious and 20% the same.  No significant improvements in physical or mental health were observed. | 356,753 generated on behalf of clients in benefits or financial assistance. |
| Cambell 2007  Longitudinal postal survey of 233 recipients of welfare advice aged 60+; time of assessment + 6 mth Follow Up. | Specialist benefits advisor working for social services. Most cases visited the elderly person at home and assessed eligibility for benefits including non-financial help such as a blue-badge parking permit. | The questionnaire incorporated a number of standard measures including SF-36, GHQ12, and self-reported Postal Barthal Index | On the SF36 scale, only two dimensions had statistically significant improvement, these being *Role limitation (physical)* and *Change in health*. There were no other significant differences noted. | Only 40 respondents at 5 months gave information regarding financial benefits and, of these, 26 (65%) had increased their benefit provision. No difference was noted between those who gained financial benefits and those who did not |
| Citizens Advice, 2010  Discussion | 690,000 clients presented to Citizens Advice Bureau 2009-10 with benefit and tax credit issues. Benefit and tax credit problems rose by 21% compared to 08-09. Jobseekers allowance advice increased 40%. |  |  | Reports Glasgow study – benefits to local economy + Brighton & Hove study. |
| Clarke 2001  Mixed method evaluation of the Family Welfare Association's Well-family service. | Family Welfare Association's Well-family service is a social support service based in primary care. Employs Family support coordinators (paid) who provide a single-door advice, information and support service in GP practices or health centres.  Counselling, liaison, advice, advocacy | Questionnaire to users and GPs | Outcomes identified by users included: prevented problems escalating; feeling more in control; fewer visits to GP and reducing or avoiding taking anti-depressants,  GPs identified benefit of referring patients to someone with more time to deal with complex problems - noted own lack of expertise in welfare rights. | Better relationships  [Note reference to NHS Plan 2000 which included proposal to provide social work services in primary care for older people] |
| Connor et al. 1999  Review examining effect of improvement in income | Tax plans, daycare subsidies, job placement services, housing allowance, monthly cash allowance interventions for low income families (majority of studies) released prisoners, 1 study lottery winners, 1 people with mental illness | Re-offending rates in prisoners, housing allowances, health effects – health status, medical usage, mental health index | New Jersey experiment - no significant impact on chronic illness, hospital days, work lost, physician visits, days in bed, days not working, illness lasting more than 3 months, illness interfering with work. Payments associated with altered patterns of medical care usage (no details). Extra income used in same way as pre-experimental income – small improvement in housing and material standard of living.  Rural income experiment – no sig effect on medical care utilisation or health status, measures of psychological wellbeing found scattered and inconsistent effects  Gary income experiment – sig improvement in birth weight in groups with 3 risk factors  Seattle/Denver – decreased hospital days for men, improved mental health index for wives, reduced duration of chronic illness for female family heads. Authors concluded payments did not affect health significantly.  Mental health study in Canada, no clear diff intervention-control groups (but small sample size) | Income maintenance was reported to moderately decrease workforce participation, no impact on marital stability, education |
| Coppell et al. 1999  Mixed method evaluation of welfare rights service | Welfare rights service in a health centre, employed welfare rights advisors |  |  | 40% of clients were found to be owed money. Both one off payments and weekly amounts were gained |
| Craig 2003  Qualitative evaluation of impact of additional benefit income for older people. | All respondents had received additional income from advice agencies, including GP practices. |  | Extra expenditure largely confined to five areas:  Essentials - food, clothing, utilities  Mobility - cars, buses, telephone  Goods and services - gardeners, handymen, cleaners, window cleaners  Lumpy items - mobility scooter, down-payment for disability car, link to local alarm system, bedding, hoover, fridge, doorbell,  Personal - presents for grandchildren | Dimensions of citizenship  Independence  Participation  Identity |
| Cullen et al. 2004  Discussion | 100 Citizens Advice Bureau mental health projects. Some bureaux report as many as 50% of clients have a mental health problem. Help provided varies; most generalist advice, bulk of which benefits and debt, specialist help for benefit reviews and appeals. Also encompasses advocacy work at outreach locations, in secure and acute units |  | By tackling core issues of discrimination and people’s need for support clients’ recovery can be facilitated and they can move forward with their life. When unwell, large numbers of people with mental health problems are on their own, they need support to cope with illness and help to deal with benefit system, debts, social activities and moves in to work. |  |
| Day et al. 2008  Qualitative evaluation of the Money advice outreach pilot projects | 22 outreach projects to deliver advice in over 100 locations, working with partner organisations – family support services, housing support services, justice system organisations, community finance organisations, other community based organisations.  Most common problems consumer credit debt, difficulties paying household bills.  Advice via face to face sessions, average 2 maximum 6. Supplemented by a mix of postal, telephone or drop in contact. Two clients referred on. Cases considered open for typically several months up to max of 6 months. | Views/perceptions via individual interviews | Reported reduced levels of personal stress. Perception of relief at receiving advice. Small number of cases money advice reportedly directed avoided losing their home. | Levels of personal finance improved reportedly for most, circumstances remained difficult in absolute terms. Around a third said they felt better able to budget and prioritise. |
| De Souza 2007  Cross-sectional evaluation of rehabilitation programme | Pappworth early rehabilitation programme: this is a 3-9 month residential programme aimed at the unemployed on incapacity benefit who express a desire to work. It includes work on dexterity, life-skills and stamina. | Coming off IB benefit. | No statement of whether health improved for those who found work. | Around 50% of those who'd been unemployed and claiming IB for 2 years were able to return to work or to seeking work. Good outcomes compared with the standard for those on IB. 274 people attended for a preliminary interview, of which 107 subsequently started a full rehab programme. Eighty-seven were male and 20 female. Half had been unemployed for more than two years. Ninety-four completed the programme, of whom 53 had gained employment, 33 were ‘work ready’ and four were doing voluntary work. At long-term follow-up, 52 were employed, 12 were in voluntary work, and 7 had retired on medical grounds However, there is no control or randomisation so caution is required in interpretation. |
| Dobbie & Gillespie, 2010  Review | Financial inclusion is a complex intervention. Financial inclusion is not only about access to benefits and income maximisation but also debt advice, financial awareness and financial capability.  All papers included advice provision. 2 studies in addition awareness raising sessions, money management guidance, development activities. 7 advice in primary care, one social services, two other advice provider premises, 1 outreach +primary care, others mix of methods including telephone advice, home visits. |  | Investment in financial inclusion is recent in the NHS initiatives are in early stages and few evaluated for impact on health. Mental health benefits of financial inclusion activity are area where research has shown clear benefits. Wider impact of addressing stress/anxiety needs exploring, people with anxiety disorders are at greater risk of developing a number of physical health conditions such as Coronary Heart Disease. Benefits of benefits advice on mental health and socio-economic wellbeing has been underplayed. | Little need to conduct additional work to determine whether welfare rights advice has a financial effect. |
| Doncaster PCT 2008  Mixed method evaluation of Healthy Living Centres | Healthy Living Centres attached to GPs, Schools, Citizens Advice Bureau offices, Leisure Centres, and so on. Large range of help provided: Referral to primary health care, NHS walk-in, dentistry, mental health services, counselling, holistic and complementary therapy, substance misuse and addiction, sexual health, family planning, food co-ops, physical activity, sports, credit unions  Advice on nutrition and diet,  Advocacy and legal advice, outreach work. Advice on benefits. |  | Some anecdotes of improved health. In the qualitative data, there is evidence that increased benefit cash was spent on items such as heating; this is plausibly linked to health. | Via GP surgeries, Doncaster Citizens Advice Bureau reclaimed about £90K [pounds Sterling] for clients. |
| Dowling 2003  Systematic review - effectiveness of financial benefits in reducing inequalities in child health | Review of RCTs of income supplementation programmes  3 studies fully met criteria but only one directly addresses question (Huston 2001) |  | Lack of high quality experimental evidence to adequately assess effectiveness of financial benefits in reducing inequalities in child health. |  |
| Ennals 1993  Discussion paper, | Role of GPs in providing advice re benefits. |  | The factor which binds health and social security together is the characteristics of GP pts – correlation between areas of high deprivation and high GP workload. Many aspects of benefit system linked to ill health and disability. | AA worth £37 per week, acts as a passport to other benefits (income support/housing) which could lead to additional £15-53 pw. |
| Finch 1993  Cross-sectional | Citizens Advice Bureau worker attending a GP surgery twice a week.  Employment, family, personal issues, tax and debt enquiries tended to predominate. 20% were repeat enquiries |  |  |  |
| Fruin 2008  Cross-sectional | Those with poor HAQ scores were contacted by a Citizens Advice Bureau advisor; benefit check; help to claim. | Was HAQ score effective in identifying those who were entitled to claim and not doing so? | No health data. | 48/86 already on benefits, although 8 advised to have review. 38 not in receipt; of these, 29 (63%) awarded benefits. Amount awarded from £14.98 to £70.76 per week. High level of satisfaction with service. |
| Galvin et al. 2000  Mixed method evaluation | Citizens Advice Bureau service in GP surgeries, types of consultation ranged from straightforward requests for information to complex problems. | Views of users, referrers and advisers |  |  |
| Gillespie et al. 2007  Mixed method evaluation of 11 Money Advice for Vulnerable Groups projects | 5 projects linked to Pathways to Work and Working Neighbourhoods, 4 targeted specific groups (lone parents, learning disabled, young people, prisoners), 2 projects focussed on developing existing services.  Approaches found to be effective – allowing time for people to make choices, working with service user groups or support workers, informal approaches building a relationship, leaflets briefings and written information.  Home visiting perceived as a valuable service. Option of accessing service via text. Telephone helpline was perceived as being anonymous and easier to talk. Most reported receiving written information.  Staff time included mostly advice work but also delivering training and talks and awareness raising activities. | Document review, interviews with staff, questionnaires, focus groups, fieldwork with users, data analysis | 46% of interviewees said accessing money advice and being provided with appropriate support had improved their mental health and wellbeing. Reduced stress or feelings of anxiety were improvements most frequently cited. 16% said dealing with financial worries had improved their relationships. | Most common outcome regarding debt was renegotiation of payments. 20% of users cited financial education and support with money management as outcome. Benefits claimed for one in five interviewees. All services generated additional income for clients. Factors affecting people’s confidence in managing financial affairs were complex. More than half reported they felt more confident in money management, many as they were now able to meet financial demands due to the higher income. More than half reported high level of confidence and understanding of cost of credit/debit. Quarter expressed very low levels. |
| Greasley & Small 2005  Qualitative evaluation | Welfare advice service | Views of workers | Workers perceived a link between dealing with social and economic problems and reducing stress and anxiety |  |
| Greasley & Small 2002  Review | Majority of issues dealt with by advice workers in GP surgeries relate to welfare benefits (range from 39% to 80% across studies) emphasis on DLA and Attendance Allowance. Wide range of other enquiries reported. | Income generated  Nottingham Health Profile  SF36  Hospital Anxiety and Depression Scale  Measure Yourself Medical Outcome Profile | 3 studies evaluated impact on health and well-being.  Veitch – trend towards improvement but not sig.  Abbott and Hobby 99/2000 – as above.  Emanuel and Begum – reduction in anxiety and depression on HADS but not sig, also MYMOP not sig.  3 studies evaluated impact on use of health services.  Emanuel and Begum – no difference before and after advice for users of service vs. non users  Abbott and Hobby – no difference before and after advice for users who gained income vs. those who gained no income, but those who gained no income increased prescriptions and practice nurse contacts compared to those who gained income.  Abbott and Davidson – no sig diff | Significant numbers of users not claiming or under claiming benefits (15 to 70%). People with mental health problems a particular group under-claiming in 2 studies. |
| Greasley 2003  Cross-sectional evaluation of the Health plus project | Health Plus Advisors (HPAs) provided advice for 2484 patients. 6 Advise workers in 30 General Practices throughout Bradford City PCT. Patients referred by health care staff or self-refer.  Also 4 specialist advisors with more complex cases relating to debt, benefits, immigration and employment.  69% South Asian  61% Female | Questionnaires delivered at 6 & 12 months post referral - return rates very low for 717 potential returns. Includes SF36 and HADS. | 75% (99/132) felt problem was affecting their health or how they felt  81% at 6 months said advice had helped them (52/64) - 21 of these had received extra income - said this reduced anxiety and stress. |  |
| Greasley & Small  2005  Cross-sectional data analysis | Welfare advice in GP practices.  76% of users were seeking advice about welfare benefits, 14% housing issues.  30% of claims related to disability-related benefits claims. In addition issues of housing, immigration, disability, health and social services, relationship breakdowns, domestic violence. | Amount of income generated |  | Total income generated over 24 months was £2389255. One in four users benefitted financially. |
| Hanratty et al. 2007  Review of financial stress and strain in terminal cancer |  |  | Ref for financial circumstances important to Quality of Life in older age and financial strain linked to physical and mental ill health.  Widespread reporting of financial strain rather than financial stress. Paucity of info on pathways between financial stress and strain, health status and Quality of Life. |  |
| Harding et al. 2002  Cross sectional - survey of practice managers | Specialist welfare advice  Home visits offered by 51% of the services. Printed welfare advice provided in 67% of GP surgeries | Views of practice managers |  |  |
| Harding et al. 2003  Cross-sectional survey of GP practice staff | Routine consultations which included a need for welfare rights advice.  55% of respondents reported specialist welfare advice available in their practice (response rate overall 47%) |  | Estimate 15% of consultations involved welfare rights issues. 86% reported there were groups of pts less able to get advice they needed especially those with language difficulties, ethnic minorities, asylum seekers, depressed/poor mental health, elderly people, those with low level of education. Characteristics of pts with welfare rights needs were 42% significant mental health problem, 33% unemployed, 16% elderly, 47% problems with housing, 9% drug/alcohol problems, 12% debt problems. 70% of practitioners reported that there had been a mental health element to the consultation. |  |
| Hobby & Abbott, 1999  Mixed method evaluation follow up 6 and 12 months. | Citizens Advice Bureau advice sessions run in seven primary care health centres in Liverpool | Longitudinal interviews plus administration of questionnaire - unclear what | Statistically significant improvements in three aspects of self-defined emotional wellbeing: the extent to which emotional problems interfered with daily activities, vitality, and mental health. |  |
| Hobby et al. 1998  Cross-sectional survey evaluation of 26 Citizens Advice Bureau projects | Citizens Advice Bureau services in GP surgeries and health centres. | Questionnaires | 18%-81% of clients recorded as having a disability across the services. Figures for ill-health incomplete.  3 projects attempted to record health gain. Birmingham (Veitch study) trend towards improvement although not significant. Ealing – GPs reported 41% fewer visits and 41% fewer prescriptions by patients using Citizens Advice Bureau service.46% of doctors reported noticing improvement in wellbeing of the patient. 80% of patients reported improvement in their physical or mental wellbeing following Citizens Advice Bureau advice. Wear Valley – 12 of 18 staff reported service had benefited health of patients. | 9 services provided per capita rate of income raised however cannot be interpreted as not clear if worked out on basis of all clients, those receiving financial advice or those whose income increased. |
| Hoskins & Smith 2002  Cross sectional | Patients aged over 64 who appeared to be physically or mentally frail were referred by community nurses. Contacted by a welfare rights officer by telephone and offered a home visit | Unclaimed attendance allowance and linked benefits |  | 37 of 86 were not claiming the benefits they were entitled to (mostly attendance allowance). Lump sum plus recurrent benefits were gained. |
| Hoskins & Carter 2000  Review | Welfare benefits advice projects  Citizens Advice Bureau sessions in GP practices, welfare benefits advisor in a health centre, open-access welfare rights sessions in a GP practice, money advice training for primary care staff, money advice worker in GP practices. | The amount of benefit money uncovered, cost effectiveness of projects, advantages of project, number of projects, Nottingham Health Profile, SF36 | Aspects of benefit system such as incapacity benefit, DLA and attendance allowance are directly linked to health and disability.  Veitch study found improvement in health (Nottingham Health Profile) for those receiving benefit increase but not statistically significant.  Abbot & Hobby (99) study found improvements in mental and physical health in those receiving additional benefit. | All the projects uncovered substantial levels of benefit payments for clients  One study (Paris and Player) suggested health centre sessions reached people who had no previous contact with Citizens Advice Bureau. One study (Middleton) suggested clients had more complex problems. |
| Hoskins et al. 2005  Cross sectional | Patients aged over 64 who appeared to be physically or mentally frail were screened by community nurses for eligibility for attendance allowance using a form developed for the purpose. Contacted by a welfare rights officer by telephone and offered a home visit | Amount of unclaimed benefit and grants gained |  | 363 (of 630) participants and 13 relatives achieved payments |
| Jacoby, 2002  Discussion paper |  |  | Supports link between financial difficulties, stress and health. Reports studies describing 23% of debtors perceiving that they were ill requiring a consultation with a doctor. Impact particularly of credit card debt on health- indicates lack of affordability of basic necessities, individuals spend less on quality goods and services for own health, stress of owing money. Drentea & Lavrakis study (2000) - Debt/income ratio is significantly associated with worse physical health and self-reported health. The financial strain of high debt/income ratio is a stressor affecting health. None of the other credit card debt indicators associated with health. Havlik et al. study – occurrence of melanoma associated with psychosocial stress (bankruptcy and unemployment). High levels of financial strain and poor coping behaviour associated with higher levels of periodontal disease and other dental problems. Link financial problems and suicide – correlation between suicide rates and economic factors. Possibility that cause effect link works the other way round though – that ill health leads to debt problems. |  |
| Jenkins et al. 2008  Cross-sectional data analysis of British National Survey of Psychiatric Morbidity |  | Clinical Interview Schedule, Schedule for assessment in Neuropsychiatry, Alcohol Use Disorder Identification Test, self-reported debt, self-reported income | Those on low income more likely to have a mental disorder (OR 2.09 CI 1.68-2.59) relationship was attenuated after adjustment for debt, and other socio-demographic variables. Of those with mental disorder 23% were in debt (compared to 8%), 10% had utility disconnection. More debts people had the more likely they were to have mental disorder |  |
| Jones, 2009  Longitudinal study Follow Up at 6 months and 12 months | Citizens Advice Bureau, focus on clients seeking advice for welfare benefits, debt and discrimination problems. Most debt then welfare benefits. | SF36, HADS, gains from welfare benefits, management of debts, financial circumstances | At Follow Up 10 (of 40) reported that in hindsight their problems had been due to a serious health issue eg old age, accident or sudden illness). 10 traced problems back to welfare issues.  Ongoing or newly diagnosed adverse health issues impacted on a significant proportion of the sample between baseline and follow up making it unlikely that improvements in health would be reported for these participants no matter how effective the service had been.  At 6 months following advice sig improvement in SF36 Social Functioning and Mental Health in a sample of 56. At 12 months sig improvement in Vitality, Social Functioning and Mental Health in a sample of 34. Mean scores for SF36 domains (except Role Emotional) improved at both Follow Up points.  HADS (anxiety and depression scale) mean scores improved at both Follow Up points (significant at 6 months for both, at 12 months for anxiety only) in sample of 67.  Client descriptions of less anxiety, less stress, fewer worries, fewer panic and anxiety attacks, more settled, more relaxed, more in control, peace of mind. | 152 debts managed for 25 clients, mean amount of debt per client was £15,000. 21 clients received additional ongoing welfare benefit payments ranging from £55.57-£8,710. 7 received one-off benefits of £27.73-£2,184.  Of 7 parents who reported being better off or more financially secure, reported better able to spend on children’s clothes, shoes, hobbies/sport.  54 (79%) of clients reported that they were better off or more financially secure as a result of Citizens Advice Bureau advice. |
| Langley et al. 2004  Cross sectional | Rheumatology patients screened using HAQ. Contact details passed to local welfare benefits advisor (Citizens Advice Bureau worker or professional advice agency). Session provided in home/advice centre/GP practice as user requested. | Health Assessment Questionnaire.  DLA and AA applications and outcomes |  | 38% were already in receipt of benefits. 50% of those screened with HAQ were offered appts with welfare advisor. Twice as many applications for AA versus DLA were made. Successful award of benefit ranged 55%-79% in different areas. |
| Levy 2006  Cross-sectional evaluation of hospice - welfare rights advocacy service provided by social workers |  | Audit of work and workload |  | In 3 months, 244 clients seen; 221 items claimed - information on 145 showed mean benefit of £52 pw. Retrospective examination of notes showed complex cases where patients were helped with debt and other major financial problems. Work included legal advocacy. |
| Lishman-Peat & Brown no date  Mixed method evaluation of GP surgery advice sessions | Advice sessions at GP surgeries (weekly, appointment-based), pts can self-refer but in practice most referrals from GP (these referrals seen at home rather than in-surgery for self referrals). | Increased income achieved, client views | Most valued part of service being listened to (and home visits) | 91% of clients either did not know their entitlements of had not felt confident enough to make a claim. |
| Lucas 2008  Systematic review of evidence from rich countries (USA, Canada) | Additional money to poor families | Child health, child mental health, emotional state | Nine trials including more than 25,000 participants were included in this review. No effect was observed on child health, measures of child mental health or emotional state. | We can not state unequivocally whether financial benefits delivered as an intervention are effective at improving child health or wellbeing in the short term. Our conclusions are limited by the fact that most of the studies had small effects on total household income and that while no conditions were attached to how money was spent; all studies included strict conditions for receipt of payments. |
| Mackintosh et al. 2006  RCT, Follow Up 24 months | Assessment by a welfare rights officer from social services. Assistance with making claims over the following months. Session offered at home or GP surgery all participants opted for home. Repeat assessments at 6, 12 and 24 months. | SF36, HADS, activity limiting long term illness, symptoms inventory, Pittsburgh Sleep Quality Index, self reported height and weight used to calculate BMI, Dietary Inventory for Nutrition Education, Physical Activity Scale for the Elderly, smoking and alcohol consumption, Social Support Questionnaire, Self Esteem Inventory, Personal Mastery Scale, Life Events Inventory, Affordability, Standard of Living Index | Participants had poorer physical health than expected for their age, mental health scores as expected.  At 6 months mean change for most outcomes was zero.  At 12 months and 24 months also outcomes varied little from baseline in intervention group. | 58% received an award including financial and non financial (eg parking permit) |
| Marcella & Baxter, 2000  Cross sectional evaluation of advice agencies | Citizens Advice, UK libraries, local information projects and centres examining citizenship information. Services attracted users from not just immediate area but further afield. Distinction between information provision versus advice. Agencies apart from libraries offered domiciliary visits in addition to centre-based service, also would carry out representation work at courts and tribunals. Organisations encompassed paid full time and part time although number varied considerably between organisations. Most agencies also relied heavily on volunteers. Citizens Advice Bureau offer basic training programme others mix of in-house and on the job training. Welfare benefits and consumer issues (debt) biggest categories of enquiries received by CAB. A general information service reported 26% social security, 18% consumer, 14% housing, 10% employment, family 10% etc... Some geographical differences eg consumer/debt ranked first in 4 places, in Birmingham immigration forms major part of workload. Other services info on education and employment, local information. Citizens Advice Bureau use info system + textbooks and other handbooks. Range of leaflets/pamphlets available. One Citizens Advice Bureau service has website for clients to access information. All bureaux N Ireland have computers for clients to access the info database themselves. |  |  |  |
| Memel et al. 2000  Cross-sectional survey of pts with osteoarthritis + their GPs |  | Health Assessment Questionnaire, HADS, employment status, benefits received, health status | Moderate prevalence of depression and high of anxiety which the GP often did not recognise | 46% of severely disabled patients were receiving disability benefits. GPs often unaware of welfare benefits received. |
| Middleton et al. 1993  Cross sectional | Advisor on welfare rights from housing department based in a health centre. Average number of contacts 6.25 per client. |  |  | 18 of 52 clients received lump sum payments, outcome of 64 claims not known. |
| Moffatt et al 2010  Qualitative interviews with people with cancer or caring for someone with cancer who were recipients of welfare rights advice. | Welfare rights advice from three professional advisors; several referral methods including self-referral; | Additional benefit income | Health impacts not directly mentioned but some implied in relation to stress and also the ability to meet heating bills, dietary needs and so on. | 18/21 received additional benefit income:  The money was used in a number of ways and included: offsetting additional costs associated with  cancer (travel and parking costs, heating bills, dietary needs, clothing needs due to weight loss/gain); lessening the impact of loss of earnings or unemployment; providing a safety net while individuals negotiated with companies over their health insurance claims. |
| Moffatt 2008  Qualitative evaluation of welfare rights service for older people | Welfare rights service - see Mackintosh 2006 | Additional benefit income; other benefits (e.g. stay-warm and blue badge); quality of life; | Being healthier and better quality food mentioned by several participants; peace-of-mind is a strong theme. | 14/25 received financial award - median gain of £57 per week.  Impact included increased affordability of necessities and occasional expenses; increased ability to cope with emergencies; reduced stress. |
| Moffatt 2009  Discussion | None | None | None | The relationship between social exclusion and poverty is complex: data suggest that initiatives to reduce exclusion can increase income and vice versa. |
| Moffatt 2009  Qualitative interviews | Full benefits assessment plus help to claim - Newcastle Welfare Rights Service | Knowledge of benefits; take-up; impact of benefits; quality of life | Most claimants had health problems leading to eligibility to AA or DLA - money was used *inter alia* for health-related needs - | Knowledge of state entitlements was extremely low. Sixteen qualified for non-means-tested health benefits; six qualified for further means-tested state benefits. Participants could better afford essential items such as food, bills, shoes, clothes and 'one off' payments. Less stress, increased independence and better quality of life were reported. |
| Moffatt 2007  From Mackintosh 2006 RCT - Further qualitative data | RCT Mackintosh 2006 - Welfare rights advice | None | None | What are barriers to older people taking welfare benefits?  Lack of knowledge; cultural inhibitions based on habitus (roughly meaning cultural norms) - information alone will not increase uptake; autonomy-based consumer model of welfare runs counter to older people's experience - a more proactive approach is needed. |
| Moffatt et al. 2006  Qualitative data from Mackintosh RCT | Assessment by a welfare rights officer from social services. Assistance with making claims over the following months. Session offered at home or GP surgery all participants opted for home. Repeat assessments at 6, 12 and 24 months. | Views of users | Participants spoke about the benefit of having the chance to talk and having someone take an interest. Benefit of assessment being offered rather than sought. Service was described as making them feel relaxed at ease and confident about applying for benefits. | 14 of 25 reported receiving additional financial resources. Reported that the income increased affordability of necessities, provided for occasional expenses, was used as a way of dealing with emergencies and increasing savings, and eased financial worries. |
| Moffat et al. 2004  Qualitative | Citizens Advice Bureau service offered sessions in GP practices |  | Impact of financial benefit was reported as a huge relief.  Receipt of additional benefits affected social world including family relationships, work, ability to care for relative, maintain independence.  All respondents reported problems with mental health – stress, anxiety or depression. These were reported as improving or stabilising as a result of welfare advice for example pressure being lifted. Other health outcomes mentioned were sleeping better, less weight loss, less smoking, reduced high blood pressure, eating less. One mentioned phoning the doctor less, one visiting doctor less | All new financial benefits related to disability, one person in addition received help re-scheduling debts.  Non-monetary benefits were received by some following financial benefit e.g. council tax exemption, meals on wheels, adaptations to house |
| Nosowska 2004  Cross-sectional survey of patients referred to social workers at a hospice in northern England over 18 months | No intervention | Routine data plus additional data on 22 hospice patients referred to Socialorker W - how many were not claiming full benefits; how many had seen professionals but not been helped and advised to claim |  | In period before claiming, each patient met between two and five health-care professionals; information offered about Attendance Allowance only 0n 3/55 meetings: patients lost income from delay |
| Paris & Player 1993  Survey of service users | Citizens Advice Bureau  314 separate problems were dealt with by advisors on behalf of 150 attenders. Most problems social security, then health, tax and duties, housing |  | People with health problems were more likely to be entitled to unclaimed benefits | 39 of 150 attenders obtained payments |
| Pleasance 2007a)  Discussion - the ethical, legal and methodological problem of randomized trials of debt advice;  RCT evaluation | Debt advice in four Job Centres throughout UK; recruitment in four waves - problems in recruiting from general population led to that being abandoned; provider was National Debtline (not CAB); two groups of 154 - 50 week follow-up; contrast between groups reduced by non-attendance of those allocated advice and advice-seeking by those not allocated; Zelen design; | EuroQol (EQ-5D) and State Trait Anxiety Index (STAI-6). Also un-validated questions relating to debt, divorce and others. | No statistically significant difference | No significant difference in the rate at which intervention and control group respondents had resolved their debt problems at the 20-week follow-up. However, the former were significantly more likely to describe their financial position as “better” than at baseline. There was also evidence that they became more knowledgeable about their financial circumstances, more focused on dealing with priority debt, and more optimistic about their future prospects, relative to control group counterparts. These findings, though, fell short of statistical significance. |
| Pleasance & Balmer 2009  Cross-sectional analysis of survey data | No intervention | 1) Incidence of long-term illness/disability and of mental illness  2) Likelihood of link to civil law problem  3) Descriptive (guess) of direction of causation | 1) 35% of all respondents suffered civil law problem over period of three years. 17% reported a stress or Mental Health problem  Mental illness patients significantly more likely to report rights problems than those with no illness or disability  Some events seemed to lead to illness e.g. divorce and homelessness  Some mental illness often attributed to events |  |
| Pleasance et al 2007  Cross-sectional national survey |  | Frequency advice on rights sought from health professionals | Health professionals' advice was sought for about 6% of rights-type problems; 4% with health link (e.g. personal injury) and 2% had no health link at all. Authors query whether health-care professionals have right skills for this and recommend outreach rights advice services - they give examples of poor advice. |  |
| Popay 2007a  GP Survey in two inner-city areas: London and Salford |  | Frequency of social problems presented to GPs |  | Most common social problems: housing; welfare benefits; GPs would have preferred to refer on to more specific services. |
| Popay 2007b  Qualitative |  | Views and perceptions |  | Some help from GPs for social problems but of a fragmented and problematic nature. |
| Porter 1998  Discussion | North Powys Prescribing citizen's advice project |  |  | Perceived as helpful to the Practices. |
| Powell et al. 2004  Economic analysis from Langley et al study | Rheumatology patients screened using HAQ. Pts identified via hospital outpatient clinic and GPs. Contact details passed to local welfare benefits advisor (Citizens Advice Bureau worker or professional advice agency). Session provided in home/advice centre/GP practice as user requested. | Health Assessment Questionnaire  Applications for DLA and AA |  | Total unclaimed allowances was £184,382 in GP setting and £9307 from referrals from hospital setting. Benefits outweigh set-up costs |
| Reading & Reynolds 2002  Mixed methods | Citizens Advice Bureau worker attached to GP practices. Families with infant under one year of age offered service by post + recommended by health visitors. Of 23 families who used service (107 expressed an interest) 13 sought advice re welfare benefits, 10 housing, 7 consumer rights, 6 debt, 5 relationships, 4 legal, 4 employment, 4 utilities, 2 taxation. | Benefits obtained  Views of users | Benefits for mental health reported – relief of worry, depression, having advice from someone, avoiding nervous breakdown. Most women perceived that the main impact of the service was on mental health, either their own, their children’s or their partner’s. | 3 families gained welfare payments, £13,000 debt rescheduled, one professional negligence case, 3 housing transfers completed, advice given regarding other issues.  One participant reported would have been unable to afford to make the telephone calls to sort out problem. Authority of the service to sort out problems. Signposting to right service e.g. solicitor. |
| Sanderson and Mahon, 2003  Cross-sectional evaluation of support service in GP practices. | Provision of dedicated on-site support in 8 GP practices from an Advice and Support Worker (ASW) to signpost patients to specific support services and assist patient in access | Satisfaction with service. |  | Debt and benefits were discussed by most who did use ASWs. There is no detail of whether the participants gained benefits but most were satisfied with the service. |
| Sherr et al 2002  Mixed method evaluation | Health-funded advice provided in Lambeth, Southwark and Lewisham | Use of services, views of services | Health benefits not measured | Older people identified as particular beneficiaries of surgery-based provision  Level of service varied - |
| Sherratt et al. 2000  Mixed method | Citizens Advice Bureau service delivered by 3 different means. Full time officer in some practices, telephone help line for professionals to use to refer, referral to in-home service for housebound.  Drawbacks with surgery-based sessions – unfilled or inappropriate appointments, non attendance, self-referrals who could have attended the central bureau. Telephone line for referrals led to most appropriate referrals. | Use of service, views of service | More than half of referrals made to the service had physical illnesses either alone (47%) or with mental health problems (14%). Mental health problems alone 29%, 10% no health difficulties. |  |
| Smith & Patel, 2008  Mixed method evaluation of Money Advice Outreach services. | 22 money advice projects | Survey of users, impact evaluation, interviews with clients. | 5% clients a threatened loss of home was averted. 2% clients a utilities disconnection was avoided. | 35% client cases were concluded with payment plans, 9% clients debt written off.  Increase in annual income of more than 1.5 million plus £353,000 in lump sum payments. |
| Taylor 2009  Review for Financial Services Authority: |  | Data from 16 waves of BHPS. Financial capability [measured by tool constructed in this research] and psychological wellbeing [measured by BHPS e.g. GHQ] What is relationship between financial capacity and psychological wellbeing? | Indices of financial capability are significantly associated with health. Strong association between financial capability and psychological wellbeing. E.g. moving an individual from relatively low levels of FC to average FC reduces their GHQ score by about 0.65 (almost 6%), increases life satisfaction, and reduces probability of individual suffering a health problem related to anxiety or depression by 15%. | Indices of financial capability are significantly associated with gender, age, marital status, number of children, health, employment status, housing tenure and income. |
| Toeg 2003  Cross sectional | 280 patients identified as over 80 from GP lists were contacted by Citizens Advice Bureau advice worker; | Financial and non-financial benefits listed:  Attendance Allowance Income Support Social Fund  Invalid Care Allowance Housing Benefit  Council Tax Benefit Healthcare costs Charity payments  Taxi card (entitles holder to journeys for £1.50) Orange badge Transport costs  Continence advice/pads Home care  Occupational therapy Careline  Community care services Wheelchair  Housing repairs Housing Legal Other | Not measured | The findings indicate that an overall total of £137 819 was gained in increased annual income amongst the screened patients; in addition, a further £11 433 was awarded in one-off payments. A wide range of other benefits and help, including referral to other services and organisations, was gained. |
| Turley & White, 2007  Qualitative evaluation of impact of advice on debt problems | Any advice | Interviews with people identified as having a money problem | Stress experienced by those in debt frequently impacted on relationships. Most reported stress and anxiety. Report of exacerbating pre-existing health conditions such as IBS and post-natal depression. Reports of debt leading to isolation – losing touch with friends, not going out socially.  Advice described as having an impact on emotional outlook. | Debt problems rarely attributed to one isolated cause. 3 causes of debt reported , changing circumstances (employment, illness, bereavement, separation), money management, creditor behaviour.  Advice reportedly helped increase levels of knowledge and confidence about how to tackle a debt problem, felt better equipped to deal with creditors, helped financial situation by calculating realistic and affordable payment plan. For some advice had not been able to offer any practical solutions, or advice was considered impractical to follow, or financial situation had deteriorated after seeking advice. |
| Veitch, 1993  Brief report on Birmingham project | Citizens Advice Bureau caseworker in post for 21 hours per week in GP practice socially deprived area of North Birmingham. |  |  | Project secured 180,077 in extra benefits for 3042 enquirers. |
| Wiggan & Talbot 2006  Review of benefits of welfare rights advice |  |  | Impact on fuel poverty on health (Baker 01)  Isolation of pensioners increased in rural areas  Sig improvement in mental health (Caiels & Thurston) | Increased take up of benefits  Local economic development (Fraser Allander Institute) |
| Williams 2004  Review of impact of debt advice | Debt counselling |  | Stress associated with debt problems is higher than general consumer disputes.  70% of over-indebted households suffered from mental health problems (study in Finland Nykanen)  Marked negative effect of debt on physical and mental health (Ahlstrom)  Links between debt and mental illness  47% of users of debtline reported that their health had improved  Criticisms of the Abbott & Hobby study – small sample multiple factors | Reference to survey reporting 1 in 12 people in financial difficulty said the situation was having a bad effect on their relationship (Kempson 02) Avoidance of family breakdown.  Benefit in terms of preserving jobs  Debt leading to criminality  Avoiding homelessness  Credit counselling associated with a positive change in borrower behaviour (Moorhead et al 2001)  Paying off of priority creditors |
| Winder et al. 2008  Qualitative evaluation of advice to older people | Specialist social services based home visiting advice team. Advice on obtaining welfare benefits. | Interviews, 13 older people and 10 carers | Most interviewees reported that the money gave them extra choice or control over important practical aspects of their lives e.g. not having to be so careful with spending, choosing higher quality meals, being able to purchase services and equipment. For some it enhanced financial security and reduced anxiety, could be used for repairs or in an emergency. For others enabled them to go on outings. Increased options for transport reported (use of taxi, maintaining own car) enabling easier access to GP or hospital or to go on trips or social events or visit relatives. New scooter, paying for alternative therapies, released time for carers by buying in services. |  |
